# A hybrid stochastic model of folate-mediated one-carbon metabolism: Effect of the common C677T *MTHFR* variant on *de novo* thymidylate biosynthesis

**DOI:** 10.1038/s41598-017-00854-w

**Published:** 2017-04-11

**Authors:** Karla Misselbeck, Luca Marchetti, Martha S. Field, Marco Scotti, Corrado Priami, Patrick J. Stover

**Affiliations:** 1 The Microsoft Research - University of Trento Centre for Computational and Systems Biology (COSBI), Piazza Manifattura, 1, 38068 Rovereto (TN), Italy; 2 Department of Mathematics, University of Trento, Trento, Italy; 3 Division of Nutritional Sciences, Cornell University, Ithaca, New York 14853 USA; 4 GEOMAR Helmholtz Centre for Ocean Research Kiel, Düsternbrooker Weg 20, 24105 Kiel, Germany

## Abstract

Folate-mediated one-carbon metabolism (FOCM) is an interconnected network of metabolic pathways, including those required for the *de novo* synthesis of dTMP and purine nucleotides and for remethylation of homocysteine to methionine. Mouse models of folate-responsive neural tube defects (NTDs) indicate that impaired *de novo* thymidylate (dTMP) synthesis through changes in SHMT expression is causative in folate-responsive NTDs. We have created a hybrid computational model comprised of ordinary differential equations and stochastic simulation. We investigated whether the *de novo* dTMP synthesis pathway was sensitive to perturbations in FOCM that are known to be associated with human NTDs. This computational model shows that *de novo* dTMP synthesis is highly sensitive to the common *MTHFR* C677T polymorphism and that the effect of the polymorphism on FOCM is greater in folate deficiency. Computational simulations indicate that the *MTHFR* C677T polymorphism and folate deficiency interact to increase the stochastic behavior of the FOCM network, with the greatest instability observed for reactions catalyzed by serine hydroxymethyltransferase (SHMT). Furthermore, we show that *de novo* dTMP synthesis does not occur in the cytosol at rates sufficient for DNA replication, supporting empirical data indicating that impaired nuclear *de novo* dTMP synthesis results in uracil misincorporation into DNA.

## Introduction

**Figure 1:**
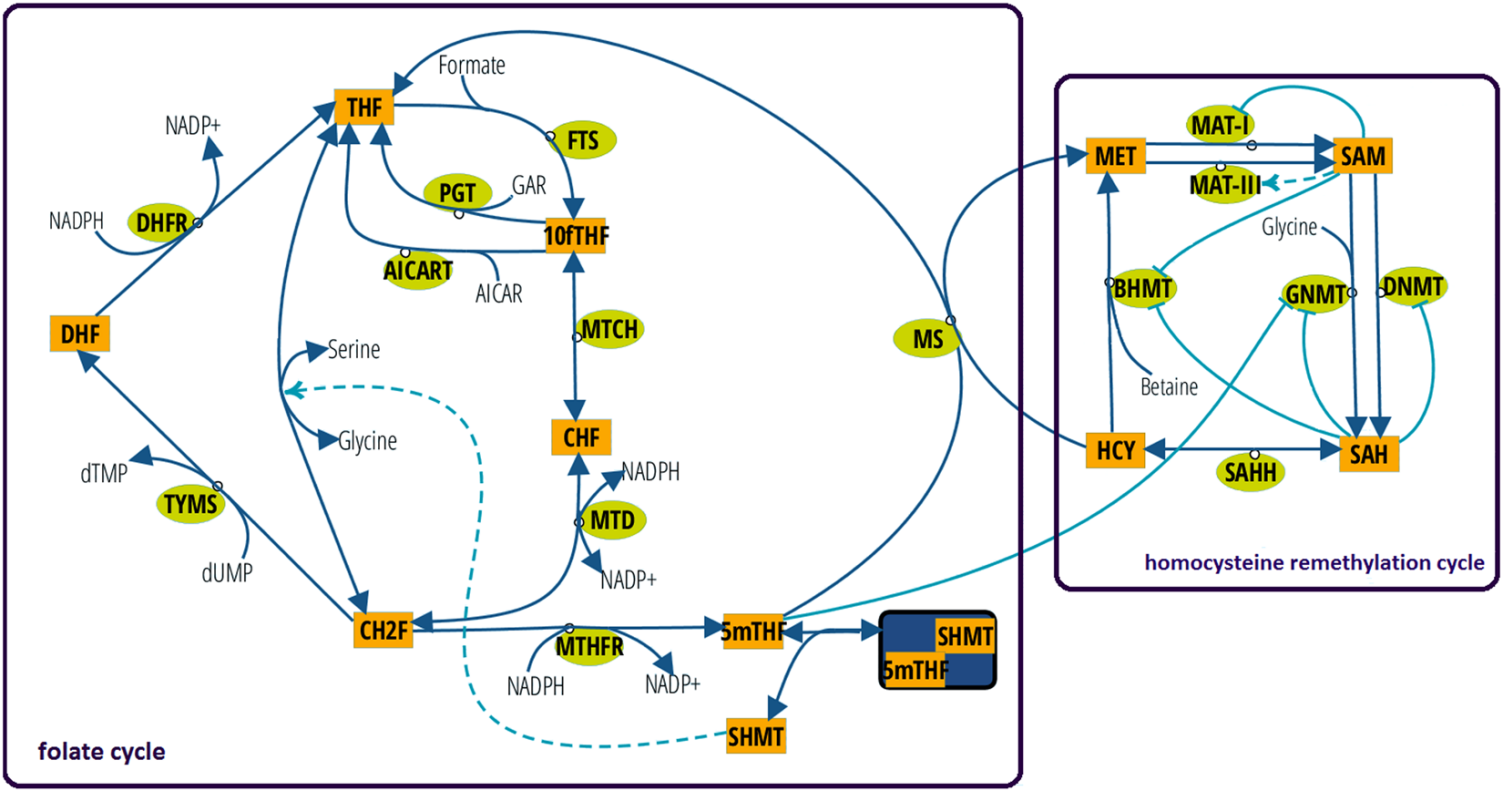
The reaction-based specification of the model according to the notation introduced in Gostner *et al*.^61^. Rectangles identify model variables, non-boxed substrates are model constants, green circles identify enzymes, dark blue arcs identify matter transformation, and light blue arcs identify regulatory events (dotted lines indicate activations and solid lines indicate inhibitions). The purple boxes indicate reactions and variables associated with the folate cycle and the homocysteine remethylation cycle, respectively.

Perturbations in folate-mediated one-carbon metabolism (FOCM) are associated with numerous pathologies including neural tube defects (NTDs)^1^, stroke^2^, colorectal and other types of cancer^3–5^. Furthermore, enzymes in FOCM have been successful targets for the development of antineoplastic pharmaceutical agents including0020methotrexate and 5-flurouracil^6^. FOCM in the cytoplasm is composed of three interconnected biosynthetic pathways, which include *de novo* thymidylate (dTMP) synthesis, *de novo* purine synthesis and homocysteine remethylation to methionine (Fig. 1). The FOCM network is sensitive to nutritional status for several vitamins that serve as enzyme cofactors (folate, riboflavin, vitamin B6 and vitamin B12) and genetic factors (coding and expression variants in folate-dependent enzymes) that can alter network outputs, including DNA synthesis, DNA repair and chromatin methylation^7–9^. Understanding the molecular basis of disease etiology has been limited by the ability to ascribe specific FOCM pathways and their biomarkers to clinical outcomes, because the pathways of FOCM are tightly interconnected^8^. FOCM complexity is manifest by: (a) competition among the pathways for a limiting pool of folate cofactors^10^, (b) long-range and indirect regulatory processes, (c) formation of multi-enzyme complexes, (d) cellular compartmentation, (e) interactions with other metabolic pathways, (f) nutritional status (g) penetrant genetic variants^9, 11^. Mathematical models have been developed to assess this complexity and gain an understanding of the cause-and-effect relationships that regulate FOCM functioning in health and disease. The overall goal is to provide an understanding of function of the entire system *in silico* that can be used to accelerate discovery and guide the design of biological experimentation.

Early models of FOCM quantified steady-state concentrations of folates, and predicted how methotrexate and 5-fluorouracil affect the rates of *de novo* purine and thymidylate biosynthesis in L1210 cells^12–15^. These models considered FOCM reactions as occurring in a common cellular compartment, described the reactions in terms of Michaelis-Menten kinetics, and investigated biological consequences of inhibiting *de novo* purine and dTMP biosynthesis. Other models have been constructed to simulate the homocysteine remethylation in liver^16–18^. Using data from experimental animals, non-trivial mathematical functions were adopted to address long-range inhibition and activation processes involving folates and other metabolites that cannot be translated into simple Michaelis-Menten equations^17^. These models can reproduce changes in metabolite concentrations in response to nutritional deficiencies and the effects of gene variants, thus corroborating human clinical and epidemiological data. These models have also integrated other pathways such as glutathione metabolism and polyamine synthesis, studying the influence of indirect effects beyond FOCM^19, 20^.

These existing FOCM models describe the continuous flux of metabolite concentrations in terms of ordinary differential equations (ODEs) with time as an independent variable^13, 20^. Sensitivity analysis is used to summarize the effects on metabolite concentrations and reaction velocities in response to changes in inputs or enzyme activities^21^. Here we present a hybrid stochastic model for simulating the FOCM dynamics where state-of-the art deterministic simulation (based on ODEs) has been coupled with exact stochastic simulation to assess metabolite variabilities in the FOCM network at steady state. A detailed description of this combined approach can be found in Methods. The deterministic approach used in isolation can only provide a rough estimate of FOCM model dynamics, because the deterministic approach is limited when enzyme substrates, such as folate cofactors, are present in the cells at low micromolar concentrations, and because reactions within the network occur randomly at discrete time points. FOCM is expected to exhibit variability (i.e. stochasticity) in its behavior^22^. Capturing system stochasticity is essential when substrate concentrations are low and limiting, but requires consideration of molecules as discrete entities, rather than describing concentrations as continuous variables through ODEs^23^. Simulation strategies that combine both deterministic and stochastic approaches can give a more accurate and more detailed understanding of FOCM network functioning and stability. In contrast to approaches based solely on deterministic simulation, these studies can be used to assess the contributions of factors such as genetic variation and nutritional status on the stochastic behavior of individual pathways within the network, thereby aiding in establishing which system inputs (i.e. nutrition) and outputs (i.e. biomarkers) are most closely associated with human health outcomes.

Dysregulation of the partitioning of one-carbon units in the form of 5,10-methylenetetrahydrofolate (CH2F) cofactors between the *de novo* dTMP biosynthesis and homocysteine remethylation pathways is believed to underlie FOCM-associated pathologies including NTDs (Fig. 1). A common variant of *MTHFR*, the C677T polymorphism, has been associated with numerous pathologies including birth defects, cancer, cardiovascular events, and other pathologies^24^. MTHFR catalyzes the FADH-dependent, irreversible conversion of CH2F to 5mTHF, which commits folate cofactors away from dTMP synthesis and towards homocysteine remethylation in the cytosol (Fig. 1). The variant results from an alanine to valine substitution in the protein that decreases MTHFR activity by decreasing its affinity for the FADH cofactor. Such substitution affects enzyme stability and hence the partitioning of folates between dTMP synthesis and homocysteine remethylation^25, 26^. Decreased MTHFR activity resulting from the polymorphism decreases 5mTHF synthesis, leading to impaired homocysteine remethylation and elevated serum homocysteine^24^. The 677 T variant is also associated with a redistribution of cellular folate cofactors; 5mTHF is the predominate form of folate in red blood cells in *MTHFR* 677CC carriers, whereas 10fTHF is the predominate form of folate in *MTHFR* 677TT carriers^27–29^. 10fTHF is less chemically stable than 5mTHF, and the MTHFR 677TT variant is associated with lower folate status^30^ and higher folate requirement^31^. Recent studies suggest that the contribution of the MTHFR variant to NTD risk is due to its impact on cellular folate status, rather than impaired homocysteine remethylation^32^. Likewise, mouse models of NTDs indicate that impaired dTMP synthesis, and not homocysteine remethylation, cause folate-responsive NTDs^33–35^. Reed *et al*. investigated the consequences of the *MTHFR* C677T polymorphism, assuming 70% enzyme activity for heterozygote and 30% enzyme activity for homozygote, in comparison to CC homozygotes (which was set to 100% activity) using parameters for folate monoglutamates, which are not the physiological form of folate cofactors in cells. Under these conditions, the variant allele decreased concentrations of 5mTHF and SAM and increased the concentrations of homocysteine, SAH, and rates of dTMP and purine biosynthesis^21^, which is inconsistent with current understanding of biochemical changes associated with NTD risk. The effect on the redistribution of folate cofactors towards 10fTHF that is associated with the 677 T variant, or its impact on other pathways within the network, was not reported^21^.

**Figure 2:**
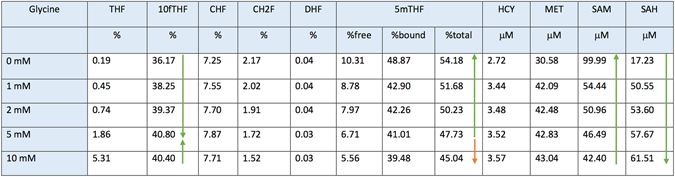
Steady states for five different values of glycine (folate cycle in % and the homocysteine remethylation cycle in μM). Where possible, a trend arrow is provided on the right to show the experimental outcome observed in Herbig *et al*.^28^. All the trends are consistent with literature (green arrows) except for the total % of 5 mTHF in the glycine range 5–10 mM (red arrow).

Here, we studied the partitioning of CH2F, a cofactor for both homocysteine remethylation and *de novo* dTMP biosynthesis^36–38^, and the effects of known genetic and nutritional variables that impact movement of CH2F through the network. Existing models are limited by adopting kinetic parameters determined from the use of folate monoglutamate substrates^19, 20^. Folate polyglutamates are the functional form of folate cofactors in cells and have much higher affinity for their respective FOCM enzymes than the corresponding monoglutamate forms of folate^39, 40^. Therefore, we updated the parameters in the deterministic model to include the physiologically-relevant polyglutamate forms of folate cofactors and demonstrate that it faithfully recapitulates existing data in the literature (see Methods, Model Validation and Fig. 2). The decisions for selecting individual enzyme kinetic parameters for this model were driven by: 1) available data for physiologically relevant polyglutamate forms of the folate cofactors derived from characterization of mammalian enzymes, and 2) data from human models, specifically L1210 cells, because of the richness and quality of the data used to derive kinetic parameters. Given the high conservation of folate enzymes among mammals, our model could be applied to mammalian systems in general, even though we are not proposing a completely homogeneous model with respect to species.

We were able to identify key nodes in the network of Fig. 1 that exhibit high degrees of stochastic behavior, including the influence of nutrient status and genetic variation on stochasticity through simulations. We explored the impact of the *MTHFR* C677T polymorphism and its interaction with folate status on partitioning of CH2F within the network, including its impact on *de novo* dTMP biosynthesis to understand the etiology of NTDs. The results of the computational model provide evidence that the rates of *de novo* dTMP synthesis as currently modeled in the cytosol are insufficient to support DNA synthesis in S-phase in mammals, accounting for uracil misincorporation into DNA that occurs in folate deficiency and in mouse models of NTDs.

## Results

### The effect of folate status and the MTHFR polymorphism on pathways affecting NTD risk

In the current model, MTHFR activity was decreased to model the effect of the *MTHFR* C677T polymorphism. In addition, a two-fold increase in MTHFR activity was modeled to examine whether there was a dose-response relationship between MTHFR activity and various readouts of the network. This model shows that 5mTHF levels decrease as MTHFR activity decreases, reflecting the effects of the *MTHFR* C677T polymorphism (Table 1). The current model also recapitulates the biological observation that decreased MTHFR activity results in accumulation of 10fTHF and THF (Table 1). Inclusion of 10fTHF in the folate distribution is a strength of the current model in that it allows for estimation of the effect that perturbations to the system have on accumulation of this unstable form of folate, which likely accounts for the decreased folate status linked to NTDs in carriers of the polymorphism^30, 32^.

**Table 1:** Distribution of folate (in percentage of total folate) for different levels of MTHFR activity (ranging from 2x to 0.3x of wild type). CC, CT and TT refer to the C677T polymorphism.

%	THF	10fTHF	CHF	CH2F	DHF	5mTHF		
						free	bound	total
MTHFR x2	0.25	14.43	2.86	0.79	0.01	33.45	48.20	81.65
MTHFR x1 – CC	0.70	39.24	7.69	1.93	0.04	8.07	42.34	50.42
MTHFR x0.7 – CT	1.59	48.76	9.45	2.18	0.04	3.37	34.61	37.98
MTHFR x0.5	3.13	54.53	10.51	2.32	0.04	1.86	27.60	29.46
MTHFR x0.3 – TT	8.12	58.72	11.26	2.40	0.04	0.90	18.56	19.46

SAM and SAH levels vary markedly with changes in MTHFR activity (SAM levels decrease and SAH levels increase by more than 50% when comparing the “CC” model to the “TT” model), with a SAM/SAH ratio around one being achieved in CC homozygotes compared to 0.24 in TT homozygotes (Table 2). Although methylation potential, otherwise known as the SAM/SAH ratio, changes markedly with varying MTHFR activity, homocysteine concentrations appear relatively insensitive to changes in MTHFR in this model (Table 2), inconsistent with known effects of the *MTHFR* TT genotype in elevating serum homocysteine levels^41^. However, in this model the lack of elevation in homocysteine due to the *MTHFR* C677T polymorphism reflects that the model represents a closed system leading to intracellular conversion of cellular homocysteine to SAH as opposed to export of homocysteine into the circulation (Table 2).

**Table 2:** Concentrations of model variables (in µM) for different levels of MTHFR activity (ranging from 2x to 0.3x of wild type). CC, CT and TT refer to the C667T polymorphism.

µM	THF	10fTHF	CHF	CH2F	DHF	5 mTHF			Unbound SHMT	HCY	MET	SAM	SAH
						free	bound	total					
MTHFR x2	0.04	2.58	0.51	0.14	0.00	5.97	8.60	14.57	0.40	3.10	37.99	78.41	31.01
MTHFR x1 – CC	0.12	7.00	1.37	0.34	0.01	1.44	7.56	9.00	1.44	3.47	42.44	51.34	53.26
MTHFR x0.7 – CT	0.28	8.70	1.69	0.39	0.01	0.60	6.18	6.78	2.82	3.67	43.00	34.87	68.98
MTHFR x0.5	0.56	9.73	1.88	0.41	0.01	0.33	4.93	5.26	4.07	3.78	42.59	26.72	77.42
MTHFR x0.3 – TT	1.45	10.48	2.01	0.43	0.01	0.16	3.31	3.47	5.69	3.89	41.87	20.47	84.29

The activity of each enzyme in the FOCM network as predicted by the current model indicates that accumulation of 10fTHF resulting from decreased MTHFR activity is due to two factors: (1) an increased flux through both the 10fTHF synthetase activity leading to increased synthesis (FTS), and (2) an increased flux through the cyclohydrolase/dehydrogenase activity of MTHFD1 which converts CH2F to CHF to 10fTHF (MTCH, MTD activities, respectively, Table 3). Interestingly, the accumulation of 10fTHF does not affect flux through the enzymes that use 10fTHF as a co-factor for *de novo* purine synthesis (PGT, AICART; Table 4). This is consistent with empirical experimental findings that increasing cellular levels of 10-formytetrahydrofolate dehydrogenase, which consumes 10fTHF, do not affect *de novo* purine synthesis^42^. Flux through the *de novo* dTMP synthesis pathway increases with decreasing MTHFR activity, consistent with empirical studies indicating these two pathways compete for CH2F (Table 4, columns DHFR, TYMS and SHMT)^43^. These findings are in agreement with empirical data showing that the TT polymorphism results in an increase in CH2F available for dTMP synthesis as indicated by isotope tracer studies in humans^26^.

**Table 3:** Fluxes of the reactions catalyzed by the enzymes FTS, MTCH, MTD, MTHFR and MTR and the binding of 5mTHF and SHMT for different levels of MTHFR activity (ranging from 2x to 0.3x of wild type). CC, CT and TT refer to the C667T polymorphism.

µM/h	FTS	MTCH		MTD		MTHFR	MTR	Unbinding of SHMT and 5mTHF
		10fTHF → CHF	CHF → 10fTHF	CHF → CH2F	CH2F → CHF			
MTHFR x2	13,101.4	332,703.8	330,313.4	37,733.2	35,342.8	26.8	26.8	17,035.2
MTHFR x1 - CC	23,582.5	756,270.3	744,706.4	89,938.7	78,374.9	21.6	21.6	14,964.5
MTHFR x0.7 - CT	31,430.7	884,173.6	864,936.0	106,222.3	86,984.7	16.0	16.0	12,231.1
MTHFR x0.5	36,054.6	954,589.4	930,806.0	115,378.8	91,595.3	11.7	11.7	9,754.6
MTHFR x0.3 - TT	39,755.7	1,002,680.1	975,243.2	121,653.1	94,216.2	7.1	7.1	6,559.7

**Table 4:** Fluxes of the reactions catalyzed by the enzymes PGT, AICARFT, DHFR, TYMS, SHMT and the unbinding of 5mTHF and SHMT for different levels of MTHFR activity (ranging from 2x to 0.3x of wild type). CC, CT and TT refer to the C667T polymorphism.

µM/h	PGT	AICARFT	DHFR	TYMS	SHMT		Binding of SHMT and 5 mTHF
					CH2F → THF	THF → CH2F	
MTHFR x2	4,406.3	6,304.6	113.5	113.5	2819.8	569.7	17,035.2
MTHFR x1 - CC	5,268.8	6,749.8	263.4	263.4	15,646.6	4,367.7	14,964.5
MTHFR x0.7 - CT	5,388.7	6,804.3	295.0	295.0	31,989.8	13,063.2	12,231.1
MTHFR x0.5	5,442.7	6,828.4	312.3	312.3	47,127.3	23,667.8	9,754.6
MTHFR x0.3 - TT	5,475.7	6,843.0	322.1	322.1	66,523.2	39,415.5	6,559.7

5mTHF binds to and is a potent inhibitor of SHMT and glycine *N*-methyltransferase (GNMT). Decreased MTHFR activity, which lowers cellular 5mTHF levels (Table 2), increases the flux through the SHMT1-catalyzed reaction in the direction of serine catabolism to glycine (Table 4). This reflects the depletion of intracellular 5mTHF as well as glycine, resulting from increased GNMT activity that results from 5mTHF depletion (Table 5). Methionine synthase (MTR) flux is highly sensitive to MTHFR genotype reflecting its dependence on availability of its substrate 5mTHF that is generated by MTHFR (Table 3).

**Table 5:** Fluxes of the reactions catalyzed by the enzymes MTR, BHMT, MAT-I, MAT-III, GNMT, DNMT and SAHH for different levels of MTHFR activity (ranging from 2x to 0.3x of wild type). CC, CT and TT refer to the C667T polymorphism.

µM/h	BHMT	MAT-I	MAT-III	GNMT	DNMT	SAHH	
						SAH → HCY	HCY → SAH
MTHFR x2	145.9	110.3	62.4	119.6	127.4	264.6	91.9
MTHFR x1 - CC	161.0	123.0	59.6	370.9	87.2	285.2	102.6
MTHFR x0.7 - CT	168.3	127.9	56.3	593.3	59.6	292.4	108.2
MTHFR x0.5	172.1	129.3	54.4	723.1	45.6	295.2	111.5
MTHFR x0.3 - TT	175.5	129.8	52.7	830.4	34.7	297.1	114.5

The MTHFR C677T variant affects both CH2F partitioning (between homocysteine remethylation and dTMP biosynthesis) and intracellular folate concentrations; the 677 T variant lowers intracellular folate levels. Therefore, the impact of this variant on FOCM was modeled at two different levels of folate (Tables 6–10) (folate replete conditions, 18 μM, and low-folate conditions, 9 μM) to understand how the variants function within the FOCM network as a function of folate cofactor availability. The results demonstrate that the changes in the percentage of 5mTHF (as well as other major one-carbon forms of folate) are more pronounced in the TT genotype than in the CC genotype when cellular folate levels are decreased (Table 6). The percentage of 5 mTHF in CC homozygous does not change in the folate replete and deficiency states, whereas the accumulation of 5 mTHF in TT homozygotes differs between the deficient and replete states (Table 6).

**Table 6:** Distribution of folate (in percentage of total folate) for replete (18 µM) and low (9 µM) levels of total folate and for the CC and TT case of the C667T MTHFR polymorphism.

%		THF	10fTHF	CHF	CH2F	DHF	5 mTHF		
							free	bound	total
low folate	CC	1.27	38.48	7.46	1.65	0.03	4.98	46.13	51.11
	TT	4.24	49.75	9.45	1.68	0.03	0.93	33.91	34.84
replete folate	CC	0.70	39.24	7.69	1.93	0.04	8.07	42.34	50.42
	TT	8.12	58.72	11.26	2.40	0.04	0.90	18.56	19.46

**Table 7:** Concentrations of model variables (in µM) for replete (18 µM) and low (9 µM) levels of total folate and for the CC and TT case of the C667T MTHFR polymorphism.

μM		THF	10fTHF	CHF	CH2F	DHF	5 mTHF			Free SHMT	HCY	MET	SAM	SAH
							free	bound	total					
low folate	CC	0.11	3.43	0.67	0.15	0.00	0.44	4.12	4.56	2.55	3.73	42.84	30.34	73.61
	TT	0.38	4.44	0.84	0.15	0.00	0.08	3.03	3.11	10.00	3.95	41.35	17.43	87.79
replete folate	CC	0.12	7.00	1.37	0.34	0.01	1.44	7.56	9.00	1.44	3.47	42.44	51.34	53.26
	TT	1.45	10.48	2.01	0.43	0.01	0.16	3.31	3.47	5.69	3.89	41.87	20.47	84.29

**Table 8:** Fluxes of the reactions catalyzed by the enzymes FTS, MTCH, MTD, MTHFR and MTR for replete (18 µM) and low (9 µM) levels of total folate and for the CC and TT case of the C677T MTHFR polymorphism.

µM/h		FTS	MTCH		MTD		MTHFR	MTR
			10fTHF → CHF	CHF → 10fTHF	CHF → CH2F	CH2F → CHF		
CC	replete folate	23,582.5	756,270.3	744,706.4	89,938.7	78,374.9	21.6	21.6
	low folate	22,583.6	427,359.0	415,960.1	48,054.6	36,655.7	13.8	13.8
absolute difference (% of replete folate)		4.2	43.5	44.1	46.6	53.2	36.5	36.5
TT	replete folate	39,755.7	1,002,680.1	975,243.2	121,653.1	94,216.2	7.1	7.1
	low folate	33,618.2	529,771.1	507,690.2	59,371.2	37,290.2	4.2	4.2
absolute difference (% of replete folate)		15.4	47.2	47.9	51.2	60.4	41.2	41.2

**Table 9:** Fluxes of the reactions catalyzed by the enzymes PGT, AICARFT, DHFR, TYMS and SHMT and the binding/unbinding of 5 mTHF and SHMT for replete (18 µM) and low (9 µM) levels of total folate and for the CC and TT case of the C677T MTHFR polymorphism.

µM/h		PGT	AICARFT	DHFR	TYMS	SHMT		Binding of 5 mTHF and SHMT	Unbinding of 5 mTHF and SHMT
						CH2F → THF	THF → CH2F		
CC	replete folate	5,268.8	6,749.8	263.4	263.4	15,646.6	4,367.7	14,964.5	1,4964.5
	low folate	4,711.3	6,473.4	117.8	117.8	18,535.4	7,268.2	8150.7	8150.7
absolute difference (% of replete folate)		10.6	4.1	55.3	55.3	18.5	66.4	45.5	45.5
TT	replete folate	5,475.7	6,843.0	322.1	322.1	66,523.2	39,415.5	6,559.7	6,559.7
	low folate	4,943.9	6,593.4	120.0	120.0	73,586.0	51,629.1	5,992.1	5,992.1
absolute difference (% of replete folate)		9.7	3.6	62.8	62.8	10.6	31.0	8.7	8.7

**Table 10:** Fluxes of the reactions catalyzed by the enzymes BHMT, MAT-I, MAT-III, GNMT, DNMT and SAHH for replete (18 µM) and low (9 µM) levels of total folate and for the CC and TT case of the C677T MTHFR polymorphism.

µM/h		BHMT	MAT-I	MAT-III	GNMT	DNMT	SAHH	
							SAH → HCY	HCY → SAH
CC	replete folate	161.0	123.0	59.6	370.9	87.2	285.2	102.6
	low folate	170.3	128.8	55.3	664.0	51.8	294.0	110.0
absolute difference (% of replete folate)		5.8	4.7	7.3	79.0	40.5	3.1	7.2
TT	replete folate	175.5	129.9	52.7	830.4	34.7	297.1	114.5
	low folate	177.4	129.8	51.8	884.4	29.4	297.9	116.3
absolute difference (% of replete folate)		1.1	0.0	1.8	6.5	15.2	0.3	1.6

Fluxes through FOCM pathways are affected by both the *MTHFR* C677T polymorphism and folate levels. The most sensitive pathways to folate deficiency are the MTCH and MTD activities of MTHFD1, MTHFR, MTR, DNMT, DHFR, and TYMS (Tables 8–10, row showing absolute flux differences between folate levels). Flux through the dTMP synthesis pathway (DHFR and TYMS) is highly sensitive to folate status for both the MTHFR CC and TT genotypes (Table 9), with the TT homozygotes being the most sensitive. Flux through GNMT was also highly sensitive to folate status in the CC homozygotes, whereas GNMT flux in TT homozygotes was insensitive to folate status (Table 10). Similar but less pronounced effects were seen for flux through SHMT (Table 9).

To understand if *MTHFR* genotype affects the stability of the FOCM network at steady state, the deterministic simulation was coupled with stochastic simulation using a hybrid simulation strategy (see Methods and Supplementary Material). Model steady states were obtained under four different conditions that differed by *MTHFR* 677 genotype (CC and TT case) and intracellular folate levels (replete and low). Interestingly, the most stable steady state (the one with lowest total sum of reaction propensities *a*_0_(*x*), see Methods for details), was the CC case with folate replete concentrations, consistent with numerous epidemiological studies associating the *MTHFR* C677T genotype with folate-related pathologies (Table 11)^43^. The enzyme that exhibited the greatest level of stochasticity in response to folate levels and/or the *MTHFR* C677T polymorphism was SHMT1 (Table S6).

**Table 11:** Total propensities obtained in four steady state conditions according to folate polymorphism (CC and TT case) and total concentration of available folate (replete, 18 µM; low, 9 µM).

*a*_0_(*x*)	replete folate	low folate	Difference (% of replete folate)
CC	1.6054 · 10^15^	2.0970 · 10^15^	30.62
TT	8.5915 · 10^15^	1.0141 · 10^16^	18.03
Difference (% of CC)	435.15	383.59	

To help comparisons, the differences between CC and TT (in % of CC) and between replete and low total folate (in % of replete folate) are indicated. A steady state that is less stable (or more noisy) than another one has higher total propensity.

### The molecular basis of uracil misincorporation into DNA

Mouse models implicate SHMT1 and impaired *de novo* dTMP synthesis in NTD risk. Impaired *de novo* dTMP synthesis causes an increase in dUMP, which when converted to dUTP causes uracil misincorporation into DNA because DNA polymerases do not distinguish between dTTP and dUTP^44^. The dTMP biosynthesis pathway enzymes (MTHFD1, SHMT, TYMS, and DHFR) are present in both the cytosol and recently have been found to function in the nucleus. In the nucleus, they comprise a multi-enzyme complex at sites of DNA synthesis that may be critical to limit rates of uracil misincorporation into DNA, but regulatory mechanisms remain unknown^45^. These enzymes are modified by the Small Ubiquitin-like MOdifier (SUMO) protein at the G1/S boundary, which permits their nuclear translocation during S-phase of the cell cycle^46^. One study showed that when nuclear translocation of this complex is impaired in a mouse model over-expressing SHMT1, rates of uracil misincorporation into DNA increased several fold^45^. In this model, SHMT1 protein levels were elevated several fold in the liver, yet its localization was restricted to the cytoplasm and nuclear SHMT1 levels were depleted compared to wild-type mice^45^. Furthermore, nuclei isolated from SHMT1 overexpressing mice exhibited lower rates of *de novo* dTMP synthesis compared to nuclei isolated from wild-type mice^45^. This suggests that *de novo* dTMP synthesis occurs when the enzymes are present in the multi-enzyme complex within the nucleus in mammals. However, no definitive experiment has been performed that identifies the relative contribution of nuclear and cytosolic dTMP synthesis to overall dTMP synthesis. Interestingly, *S. cerevesiae* do not import the dTMP synthesis pathway into the nucleus^47^.

To determine if nuclear import of the *de novo* dTMP pathway was required to meet cellular demands for dTTP during DNA replication, rates of dTMP synthesis were modeled for mammalian cells using standard Michaelis-Menten kinetics (Table 12; Table 4). Based on the number of A-T base pairs in the human genome and an 8-hour S-phase in embryonic stem cells (S-phase in L1210 cells is also 6–10 h^48, 49^), the rate of dTMP synthesis required for faithful cell replication is 7.8 µM/min (calculations are in Supplementary Material, see also Table 12)^50, 51^. In the current model, which does not account for SHMT1/TYMS/DHFR/MTHFD1 nuclear localization nor complex formation, cytosolic dTMP synthesis rates are 4.4 µM/min (Table 4, DHFR and TYMS flux, 263.4 µM/h, assuming MTHFR 677 CC genotype). This computational deficit between dTMP requirements and dTMP synthesis rates suggests that dTMP synthesis as currently modeled in the cytosol where the enzymes are not present in a complex cannot meet cellular needs. Nuclear localization and complex formation of the *de novo* dTMP synthesis complex seem to be unique to mammalian cells. In *S. cerevesiae*, TYMS is not SUMOylated and localizes to the nuclear periphery^47^. The measured rate of dTMP synthesis in *S. cerevesiae* is 1.8 µM/min^52^ (Table 12). The rate of dTMP synthesis required to replicate the *S. Cerevesiae* genome over the course of an S-phase (less than one hour^52, 53^) is 0.5 µM/min, indicating that yeast synthesize dTMP at a rate that is more than 3-fold greater than necessary for adequate dTMP synthesis (calculations are in Supplementary Material). Furthermore, in response to DNA damage, yeast increase dNTP concentrations 6–8 fold^54^ and *E. coli* increase dNTP concentrations 1.8–3.7 fold^55^, but dNTP concentrations do not increase after DNA damage in mammals^56, 57^.

**Table 12:** Cellular capacity for *de novo* dTMP synthesis in mammals and yeast at S-phase.

	Human	S. cerevesiae
Genome size	3.0 × 10^9^ bp	1.2 × 10^7^ bp
% AT	59%	61.5%
T bases needed for replication	1.77 × 10^9^ molecules	7.5 × 10^6^ molecules
Length of cell cycle	24 h	2.5 h
Length of S-phase	8 h	0.83 h
Cell volume	8 × 10^−13^ L (ES cell)	5 × 10^−14^ L
dTMP synthesis rate required to replicate genome	7.8 µM/min	0.5 µM/min
Measured dTMP synthesis rate (model outcomes)	4.4 µM/min	1.8 µM/min
Ratio of dTMP production relative to dTMP required for replication	0.6	3.6

## Discussion

Understanding the dynamics of FOCM and its responsiveness to both genetic and environmental perturbations is the key to understanding the etiology of folate-related pathologies. Computational models and related simulations permit an identification of the most sensitive reactions within the network that exhibit the greatest degree of stochastic behavior leading to variability in network outputs. Furthermore, computational models allow an understanding of how both genetics and environmental factors can enhance or repress stochastic behavior at defined locations within the network, accelerating the development of diagnostics to identify those at risk for folate-related pathologies as well as lead to the development of targeted nutritional interventions for disease prevention.

The *Shmt1* knockout mouse model (*Shmt1* ^+/−^, *Shmt1* ^−/−^ embryos) exhibits impaired *de novo* dTMP synthesis in the absence of perturbations of homocysteine remethylation. It also recapitulates risk for NTDs in humans. Specifically, the mouse model exhibits folate-responsive NTDs that occur with minimal perturbation in FOCM, and exhibit low and variable penetrance^33, 34^. In fact, most if not all, folate-related pathologies whose etiology involves interactions among genetic risk variants and nutrient exposures also exhibit low and/or variable clinical presentation. Understanding the stochastic behavior of the various reactions within FOCM that results in increased variability in FOCM network outputs is essential to understand which enzymes in the network contribute to folate-related pathologies.

Existing FOCM models rely on the limited quantity of kinetic data present in the literature, and the performance of the model will be dependent upon the kinetic parameters chosen to include in the model. Much of the available kinetic data for FOCM enzymes present in the literature was collected using the commercially available monoglutamate folate substrates, with few studies using the physiologically relevant polyglutamate forms of the cofactor. In the cell, newly transported monoglutamate folates are converted to folate polyglutamates, containing 3 to 7 polyglutamate moieties, though the action of folylpolyglutamate synthetase^58^. The polyglutamate chain (N = 3 glutamate and higher) increases the affinity of folate cofactors for many folate-dependent enzymes by one to two orders of magnitude^39, 40^. Models that include kinetic parameters derived from the use of folate monoglutamates can limit model reliability. Here we established a hierarchy of criteria to select a more homogeneous set of kinetic parameters (i.e. *K*_*m*_ and *V*_*max*_) by referring, when possible, to L1210 cells because of the richness and quality of the data used to derive kinetic parameters. Furthermore, our preference was to select kinetic coefficients generated using folate polyglutamate cofactors and purified proteins from animal models closest to humans, as the variability in kinetic parameters among mammals is much less than the differences observed between folate monoglutamate and polyglutamate cofactor substrates.

The current model was validated by demonstrating that it recapitulates empirical observations regarding the impact of intracellular glycine on behavior of the FOCM network (Fig. 2). The validated model was then used to understand how the MTHFR C677T polymorphism, a known genetic risk factors for NTDs in humans, affects FOCM. This model shows that the lower levels of 5mTHF associated with the *MTHFR* 677 T variant are accompanied by elevated levels of 10fTHF, which has been observed in animal models and in humans^27–29^ (Table 1). The model also indicates that 10fTHF accumulates in TT homozygotes as a result of increased flux through both the synthetase activity of MTHFD1 (FTS activity, Table 3), but also due to increased flux through MTHFD1 activity in the direction converting CH2F to 10fTHF (Table 3). Therefore, the model accurately predicts perturbations in FOCM that have been observed in human clinical and epidemiological studies. A recent study suggested that the risk of the *MTHFR* C677T polymorphism for NTDs was due to its known effect on lowering intracellular folate concentrations, rather than its role in providing 5 mTHF for homocysteine remethylation^32^. This model demonstrates that the *MTHFR* C677T polymorphism elevates levels of 10fTHF, which is known to be a chemically unstable form of folate that is susceptible to oxidative degradation, providing a mechanism by which the *MTHFR* C677T polymorphism depletes intracellular folate levels. Importantly, the model reported here demonstrates that the *de novo* dTMP biosynthesis enzymes are the most sensitive to low intracellular folate concentrations, with both DHFR and TYMS activities being repressed by 63% (Table 9). This finding is consistent with the finding that mouse models with impaired *de novo* dTMP biosynthesis are susceptible to NTDs in folate deficiency^34^. The hybrid stochastic simulation also reveals that both folate deficiency and the *MTHFR* C677T polymorphism create overall instability in the network (Table 11), consistent with a vast body of literature demonstrating an association of both folate deficiency and the *MTHFR* C677T polymorphism with various pathologies^24, 30^. Interestingly, SHMT1 exhibits the greatest increase in stochastic behavior as a result of the *MTHFR* C677T polymorphism (Table S6); the SHMT1 enzyme is the only FOCM enzyme that when disrupted results in folate-responsive NTDs^34^.

The primary findings of this study are that the FOCM network is destabilized as a result of folate deficiency and the MTHFR677T polymorphism, and that SHMT is the most sensitive enzyme within the network to this network instability. This finding nicely connects the MTHFR genetic variant, a known risk factor for human NTDs, and SHMT1, the only folate enzyme whose disruption results in folate-responsive NTDs in mice. Furthermore, this model predicts that *de novo* dTMP synthesis rates in mammals are about half of what is required to meet DNA replication demands for dTMP (Table 12). Although mammals contain two pathways for dTMP synthesis, the folate-dependent *de novo* dTMP synthesis pathway described here and a salvage pathway catalyzed by thymidine kinase 1, the salvage pathway activity is insufficient to meet cellular needs based on observations that folate deficiency results in elevated uracil accumulation in DNA. In mammalian cells, the *de novo* dTMP synthesis enzymes form a multi-enzyme complex that interacts with DNA replication enzymes^46^. The discrepancy between *de novo* dTMP synthesis rates required to replicate the genome and the rate of dTMP synthesis currently predicted by the model indicates that the model should be extended to include multi-enzyme complex formation and substrate channeling in the nucleus to model more accurately determinants of FOCM and dTMP synthesis. The inclusion of the dTMP multi-enzyme metabolic complex in the model is expected to limit substrate diffusion and increase the rate of dTMP synthesis.

## Methods

### Description of the Model and of the Simulation Techniques

The model was constructed as a closed system using the subset of reactions that describe the FOCM pathways and homocysteine remethylation in cytoplasm^19^ (Fig. 1). For the simulation, we employed a hybrid stochastic approach using deterministic simulation to compute the initial phase of the dynamics until a model steady state was reached, and then we assessed the stability of the achieved steady state by relying on exact stochastic simulation. We adopted a hybrid approach, rather than one entirely based on exact stochastic simulation^59^, because of the intensive computational effort introduced by the stiffness of the system during the simulation.

The deterministic simulation was based on ODEs, where reactions were described in terms of Michaelis-Menten equations consistent with the original model of Reed^19^ and computed using the MATLAB integrator *ode15s*, whereas parameter estimates were derived from literature or calculated by nonlinear least squares optimization. The kinetic constants were obtained from folate polyglutamate cofactors and their interaction with enzymes purified from L1210 cells where possible and otherwise from other mammalian tissue (see Supplementary Material for a detailed discussion of the model and parameter estimates).

The set of ODEs was further translated into a stochastic reaction-based model and a hybrid simulation approach^60^ was employed to quantify the level of stochasticity in the considered FOCM steady states. We refer to the Supplementary Material for a detailed description of how the ODE model has been translated to a reaction based stochastic one. According to the seminal work of Gillespie^59^, exact stochastic simulation allows simulation of each reaction event asynchronously when such reaction event is most probable to occur. To allow this, the simulation algorithm computes a propensity function *a*_*j*_(*x*) at each simulation step for each modeled reaction *R*_*j*_, where *x* is the current state of the system providing the abundances of all modeled species at the considered time. The propensity value of a reaction has a direct link with the probability of its execution, that is, reactions with higher propensity are more likely to be fired in the near future. To evaluate when the next reaction event will occur, also the total sum of propensities $\,{a}_{0}(x)=\,\sum _{{R}_{j}}{a}_{j}(x)$ is computed, because this quantity is linked to the number of reaction events occurring in the next time unit, that is, with increasing total propensity the number of reaction events per unit of time also increases. In Table 11, we provide the total sum of propensities for the considered steady states. On average, we would need to generate up to 10^15^–10^16^ reaction events per unit of time during the stochastic simulation, due to stiffness of the system. To circumvent the problem, we relied on the concept of total propensity to evaluate the stability of the steady state, by assuming that a steady state is more stable when it has a lower value of *a*_0_(*x*), that is, a lower averaged number of reaction events that can perturb the equilibrium of the steady state.

### Model validation

To validate the FOCM model, *in silico* experiments were performed to determine if the model could recapitulate empirical data generated in MCF-7 cells by Herbig *et al*.^10^ focusing on the effect of glycine on FOCM (Fig. 2). Glycine is important because, as a second substrate, it has a direct influence on the reaction catalyzed by the enzymes GNMT as well as on the reversible reaction transforming CH2F to THF catalyzed by the enzyme SHMT. The purpose is to understand how the steady state of FOCM is affected by altering intracellular glycine concentrations. This was achieved by running several model simulations starting from different glycine concentrations and comparing the corresponding steady states with empirical data from Herbig *et al*.^10^. This study examined the effect of exogenous glycine at concentrations from 0 to 10 mM on the relative distribution of folate one-carbon forms as well as *S*-adenosylmethionine (SAM) and *S*-adenosylhomocysteine (SAH) levels. In summary, the empirical data revealed that as glycine concentrations increase intracellular 10fTHF levels increase at the expense of 5mTHF levels that decrease. Furthermore, as glycine concentrations increase SAM levels are depleted and SAH levels rise. These changes were interpreted by the effects of glycine concentration driving the reversible SHMT reaction in the direction of serine synthesis^10^.

We simulated the effect of glycine on folate distribution, SAM, and SAH concentrations using the computational model for values of glycine ranging from 0 to 10 mM (Fig. 2). The trends obtained by the model simulations were in agreement with the literature (green arrows in Fig. 2), confirming the coherence between model outcomes and empirical data. We observed only one exception related to the total % of 5 mTHF at 10 mM glycine (red arrow in Fig. 2). This discrepancy could be mainly due to two reasons: (1) 10 mM glycine is an extreme and non-physiological intracellular glycine concentration that could cause pharmacological effects, (2) the large magnitude of the experimental error in the Herbig *et al*. study at this glycine concentration.

## Electronic supplementary material

Supplementary Data
