# Supplementary material for: A hybrid stochastic model of folate-mediated one-carbon metabolism: Effect of the common C677T MTHFR variant on de novo thymidylate biosynthesis
Source: Sci Rep. 2017 Apr 11;7:797. doi: 10.1038/s41598-017-00854-w (PMC5429759; doi:10.1038/s41598-017-00854-w)
Supplement: Supplementary file 1 — Supplementary Data [file 41598_2017_854_MOESM1_ESM.pdf]

Supplementary Material

"A hybrid stochastic model of folate-mediated one-carbon metabolism: Effect of the common C677T *MTHFR* variant on *de novo* thymidylate biosynthesis"

Karla Misselbeck[1,2+], Luca Marchetti[1+], Martha S. Field[3], Marco Scotti[4], Corrado Priami[1,2*] and Patrick J. Stover[3*]

[1]The Microsoft Research - University of Trento Centre for Computational and Systems Biology (COSBI), Piazza Manifattura, 1, 38068 Rovereto (TN) – Italy

[2]Department of Mathematics, University of Trento, Italy

[3]Division of Nutritional Sciences, Cornell University, Ithaca, New York 14853, USA

[4]GEOMAR Helmholtz Centre for Ocean Research Kiel, Düsternbrooker Weg 20, 24105 Kiel, Germany

[*]these authors contributed equally to this work
[+]these authors contributed equally to this work

Table of contents

1.1 THE ODE MODEL

EQUATIONS
COMPARISON WITH LITERATURE

1.2 STOCHASTIC SPECIFICATION OF THE MODEL

1.3 CALCULATIONS OF DTMP SYNTHESIS CAPACITY IN MAMMALS AND YEAST

REFERENCES

SUPPLEMENTARY FIGURES

SUPPLEMENTARY TABLES

1.1 The ODE model

The reactions considered in the model are graphically represented in Figure 1 and in Figure S1 according to the graphical notation introduced by Gostner *et al.* [1]. Hereafter we will refer to this figure for introducing model equations.

In order to be consistent with previous literature, the model has been initially defined as a set of ordinary differential equations (ODEs) as introduced below and then translated into a stochastic model as explained in Section 1.2. The model describes the folate-mediated one-carbon metabolism (FOCM, for a list of the abbreviations used, please refer to Table S1) in the cytoplasm. In this compartment FOCM connects three pathways: *de novo* thymidylate biosynthesis, *de novo* purine synthesis, and homocysteine remethylation[2].

Equations

Mathematical specifications of FOCM have been published elsewhere [3–9]. For a discussion of the changes compared to these models please refer to the next section *Comparison with literature*.

The model consists of twelve variables:

- the different forms of folate: THF, 10fTHF, CHF, CH2F, DHF, and 5mTHF;
- the enzyme SHMT;
- the complex 5mTHF: SHMT formed by binding of 5mTHF to SHMT;
- the metabolites MET, HCY, SAM and SAH.

The other substrates (*NADPH, NADP, dUMP, Serine, Glycine, GAR, AICAR, betaine and formate*) are approximated to be constant over time in agreement with previous modeling literature [5]. In terms of reaction description, constant reactants are indicated above the arrow next to the enzyme:

$$S_1 \xrightarrow{enzyme, S_2} P.$$

Due to the biological description of FOCM, our model can be divided into two connected modules, as indicated in Figure S1. The first one specifies the reactions linking the different forms of folate; we further refer to this module as the folate cycle. The second module is the homocysteine remethylation cycle, including the four metabolites HCY, MET, SAM and SAH and the six reactions associated with these. The connection between those two parts is formed by the biochemical reaction catalyzed by methionine synthase (MTR), which regenerates MET from HCY using 5mTHF as the donor of one methyl group.

Following [5], most of the enzymatic reactions considered in the model have been translated in the set of ODEs by means of Michaelis-Menten kinetics, which consider one or two different substrates S_1 and S_2.

The formula of the Michaelis-Menten kinetics with one substrate S is:

$$v(S) = \frac{V_{max}\, S}{K_m + S},$$

where V_{max} indicates the maximum rate of the considered reaction and K_m is the Michaelis-Menten constant that specifies the concentration of the associated substrate for which the rate is half-maximum. In the same way, the formula can be extended to consider two substrates:

$$v(S_1, S_2) = \frac{V_{max}\, S_1\, S_2}{(K_{m1}+S_1)(K_{m2}+S_2)},$$

where K_{m1} and K_{m2} are the Michaelis-Menten constants for the two substrates.

For a clear and unique description of the model, each reaction and each velocity is labeled with the name of the enzyme catalyzing it. For example, R_{DHFR} and v_{DHFR} define the reaction and the velocity of the transformation of DHF to THF, catalyzed by dihydrofolate reductase (DHFR). All variable initial concentrations, constant values, and parameter estimates for the folate cycle and homocysteine remethylation used in the model are listed in Table S2, Table S3, Table S4 and Table S5, respectively. All concentrations are expressed in µM, while time is expressed in hours.

For the sake of simplicity, hereafter we will present the mathematical model by focusing on reactions and their corresponding kinetic formulas. The differential equations of the model can then be derived by summing these formulas according to reaction stoichiometry. In fact, each arrow from the model visualization in Figure S1 connected to one variable corresponds to one term in the sum of the associated differential equation. For example, if we consider DHF we see that this variable is connected to one outgoing and one incoming arrow (promoted by DHFR and TYMS, respectively). Therefore, the corresponding differential equation is:

$$\frac{d\,[DHF]}{dt} = v_{TYMS}(CH2F, dUMP) - v_{DHFR}(DHF, NADPH\,),$$

where v_{TYMS} and v_{DHFR} will be defined in the following.

The Folate Cycle

The majority of the reactions in the folate cycle are unidirectional with a time-variant and a constant reactant, like R_{DHFR} :

$$R_{DHFR} : \; DHF \xrightarrow{DHFR,NADPH} THF$$

$$v_{DHFR}([DHF], NADPH) = \frac{V_{max} \; [DHF] \; NADPH}{(K_{DHF} + [DHF])(K_{NADPH} + NADPH)},$$

where squared brackets indicate the variable concentrations.

The same translation can be applied also to the following reactions:

$$R_{TYMS} : \; CH2F \xrightarrow{TYMS,dUMP} DHF, \qquad R_{AICARFT} : \; 10fTHF \xrightarrow{AICARFT,AICAR} THF,$$

$$R_{PGT} : \; 10fTHF \xrightarrow{PGT,GAR} THF, \qquad R_{FTS} : \; THF \xrightarrow{FTS,formate} 10f\,THF,$$

$$R_{MTHFR} : \; CH2F \xrightarrow{MTHFR,NADPH} 5m\,THF.$$

The only reaction with two non-constant substrates in the folate cycle is the one catalyzed by methionine synthesis:

$$R_{MTR} : \; HCY \; + 5mTHF \xrightarrow{MTR} THF + MET$$

$$v_{MTR}([HCY], [5mTHF]) = \frac{V_{max} \; [HCY] \; [5mTHF]}{(K_{HCY} + [HCY])(K_{5mTHF} + [5mTHF])}.$$

The V_{max} estimate of this reaction is the only one that has not been directly taken from literature, but rather optimized in the range 0.024 – 500 $\frac{\mu M}{h}$ from literature [6,10] to obtain the trends discussed in the main text (see Table S4).

The next subset of reactions of the folate cycle contains the three bidirectional reactions R_{MTCH}, R_{MTD} and R_{SHMT} . R_{MTCH} links 10fTHF and CHF as a bidirectional reaction with one substrate:

$$R_{MTCH} : 10fTHF \underset{MTCH}{\overset{MTCH}{\rightleftarrows}} CHF$$

$$v_{MTCH}([10fTHF],[CHF]) = \frac{V_{max,10fTHF}\ [10fTHF]}{\left(K_{10fTHF} + [10fTHF]\right)} - \frac{V_{max,CHF}\ [CHF]}{(K_{CHF} + [CHF])}.$$

The subsequent reaction R_{MTD} is a bidirectional reaction with two substrates:

$$R_{MTD} : CHF \underset{MTD,NADP}{\overset{MTD,NADPH}{\rightleftarrows}} CH2F$$

$$\begin{aligned} v_{MTD}([CHF], NADPH, [CH2F], NADP) \\ = \frac{V_{max,CHF}\ [CHF]\ NADPH}{(K_{CHF} + [CHF])(K_{NADPH} + NADPH)} \\ - \frac{V_{max,CH2F}\ [CH2F]\ NADP}{(K_{CH2F} + [CH2F])(K_{NADP} + NADP)}. \end{aligned}$$

A slight change in terms of Michaelis-Menten kinetics can be found in the glycine and serine dependent reaction between THF and CH2F. As this reaction is catalyzed by the time-dependent enzyme SHMT, its change of concentration has to be taken into account [8]. Therefore, we used here the turnover number k_{cat}, which describes the conversion of the Enzyme-Substrate complex to the product, where

$$V_{max} = k_{cat}\ [SHMT].$$

The reaction is then defined in the following way:

$$R_{SHMT} : THF \underset{SHMT,Glycine}{\overset{SHMT,Serine}{\rightleftarrows}} CH2F$$

$$\begin{aligned} v_{SHMT}([THF], Serine, [CH2F], Glycine) \\ = \frac{k_{cat,THF}\ [SHMT]\ [THF]\ Serine}{(K_{THF} + [THF])(K_{Serine} + Serine)} \\ - \frac{k_{cat,CH2F}\ [SHMT]\ [CH2F]\ Glycine}{(K_{CH2F} + [CH2F])\left(K_{Glycine} + Glycine\right)}. \end{aligned}$$

The concentration of active SHMT enzyme changes over time because in the model we consider the tight binding of 5mTHF to SHMT [8]. The corresponding reactions are modeled by mass action kinetics with rates $k_{binding}$ and $k_{unbinding}$ [11]:

$$\text{SHMT} + \text{5mTHF} \underset{k_{unbinding}}{\overset{k_{binding}}{\rightleftarrows}} \text{SHMT : 5mTHF}$$

$$v_{binding} = k_{binding}\,[5mTHF][SHMT]$$

$$v_{unbinding} = k_{unbinding}[5mTHF\!:\!SHMT].$$

Homocysteine Remethylation

The reactions responsible for homocysteine remethylation involve BHMT, DNMT, GNMT, MAT-I, MAT-III and SAHH. Such reactions are entirely taken from literature[6].

A bidirectional Michaelis-Menten function is used to model the conversion between HCY and SAH.

$$R_{SAHH}:\ HCY \underset{SAHH}{\overset{SAHH}{\rightleftarrows}} SAH$$

$$v_{SAHH}([HCY],[SAH]) = \frac{V_{max,HCY}\,[HCY]}{K_{HCY}+[HCY]} - \frac{V_{max,SAH}\,[SAH]}{K_{SAH}+[SAH]}.$$

The betaine-dependent reaction of remethylation of HCY is presented as a two substrate Michaelis-Menten equation with an additional inhibition term dependent on SAM and SAH:

$$R_{BHMT}:\ HCY \xrightarrow{BHMT,Betaine} MET$$

$$v_{BHMT}([HCY],Betaine\,) = \frac{V_{max}\ [HCY]\ Betaine}{(K_{HCY}+[HCY])(K_{betaine}+Betaine)}\ e^{-0.0021\,([SAM]+[SAH])}e^{0.0021\,(102.6)}.$$

For the conversion of MET to SAM, two reactions which are regulated by MAT-I and MAT-III, respectively, are considered. The first one is a first-order Michaelis-Menten function with a nonlinear inhibition term dependent on SAM. $R_{MAT-III}$ was fitted to a Hill equation, including also an activation term by SAM:

$$R_{MAT-I}:\ MET \xrightarrow{MAT-I} SAM$$

$$v_{MAT-I}([MET]) = \frac{V_{max}\ [MET]}{K_{MET}+[MET]}\ (0.23+0.8e^{-0.0026\,[SAM]})$$

$$R_{MAT-III}:\ MET \xrightarrow{MAT-III} SAM$$

$$v_{MAT-III}([MET]) = \frac{V_{max}\ [MET]^{1.21}}{K_{MET} + [MET]^{1.21}} \left(1 + \frac{7.2\ [SAM]^2}{K_a^2 + [SAM]^2}\right).$$

Two methyltransferases DNMT and GNMT are included in our model; both are affected by the inhibition of SAH:

$$R_{DNMT}:\ SAM \xrightarrow{DNMT} SAH$$

$$v_{DNMT}([SAM]) = \frac{V_{max}\ [SAM]}{K_{SAM}(1 + \frac{[SAH]}{K_i}) + [SAM]}$$

$$R_{GNMT}:\ SAM \xrightarrow{GNMT, Glycine} SAH$$

$$v_{GNMT}([SAM], Glycine) = \frac{V_{max}\ [SAM]\ Glycine}{(K_{SAM} + [SAM])(K_{Glycine} + Glycine)} \frac{1}{1 + \frac{[SAH]}{K_i}}.$$

Comparison with literature

The first mathematical models of FOCM were developed in the 1970s and 1980s [12–14] mainly focusing on the effect of anticancer drugs on the network. Starting from these first attempts, new models were introduced in literature to update/extend our understanding of the network based on new experimental evidence (please refer to [15] for a review of them).

The folate and homocysteine remethylation cycles considered in our model have been initially modeled separately [3,4] and have later been merged in a single model [5]. The latter has been further extended by the same authors to study different aspects of FOCM (e.g. the interplay between mitochondrial and cytoplasmic FOCM or the inclusion of the glutathione metabolism) [6–9] Following the same approach, the model herein proposed can be considered as another extension of this previously published model [5]. In the following, this model will be used as reference for the comparison with literature.

The model presented herein constitutes an update with respect to [5] according to two main aspects. The first and most important one relates to a more physiologically relevant selection of parameter estimates in modeling the folate cycle. Parameter estimates were chosen according to two criteria. First, a homogeneous set of parameters was identified by referring, when possible, to L1210 cells. We chose this cell line because of the richness of the data available for quantifying enzyme levels and for their kinetic characterization using polyglutamate substrates. The second criterion relates to the length of the glutamate chain attached to the folate. In previous models most of the parameters are estimated using the affinity of the enzymes for monoglutamates. However, polyglutamate forms are the physiologically relevant cofactors. They play a crucial role to sequester folates in the cell given their higher affinity with the enzymes if compared to monoglutamates [16]. Therefore, our preference was to select data from polyglutamate-derived coefficients. For any detail on considered cell lines and length of glutamate chain, please refer to Table S4.

The second improvement to the structure of model equations, which have been modified to add new reactions and to update other reactions by including new biochemical interactions as described below. Figure S2 displays a point-to-point comparison between the structure of our model and the one in [5]. All modifications have been highlighted by coloring the corresponding part of the network (the green color identifies unmodified reactions, orange indicates updates/novelties, black indicates not included parts). In the following we will provide a detailed list of all the modifications.

1. Our model includes two new terms to take into account that, according to [17], the rate of reaction R_{MTD} depends also on NADPH and NADP as second substrates.

2. We further included the bidirectional reaction modeling the binding/unbinding of 5mTHF and SHMT [8,11], to study the effect of the MTHFR polymorphism on the availability of unbound SHMT. This update also affected the reaction R_{SHMT}, which has been updated to consider SHMT as model variable rather than a constant.
3. We also tried to build a parsimonious model in order to reduce the problem of overfitting of the system dynamics as much as possible. In particular, a closed model was built rather than considering external input/output pathways (see MET and cystathionine in Figure S2). Moreover, the number of non-enzymatic reactions was reduced because of a high degree of uncertainty regarding their physiological significance (see the non-enzymatic reaction NE in Figure S2).
4. The inhibition of MTHFR by SAM initially proposed in [5] could not be integrated in our model, because it was modeled by a nonlinear term whose parameters have been estimated under conditions that do not apply to our modeling scenario. In fact, the estimation of this term was based on the external methionine input (included in [5], but not in our closed model, see previous point) and on some kinetic parameters which differ by an order of magnitude with respect to estimates herein considered. Moreover, following [5], the inhibition should only act when SAH<SAM, but this is not a common case in our simulations.
5. Finally, we decided not to include the reaction between 10fTHF and THF promoted by the enzyme FTD, because we observed that the strong inhibition of FTD by 10fTHF discussed in [18] makes the effect of this reaction in the considered steady states negligible.

1.2 Stochastic specification of the model

In addition to the ODE specification, the model has been translated to a stochastic reaction based formulation. This was achieved by scaling all metabolite and enzyme concentrations, as well as Michaelis-Menten constants, to number of molecules instead of concentrations. If we consider, for example, the concentration $[THF] = 0.12$ µM, the corresponding number of molecules $\#THF$ is

$$\# THF = [THF] \cdot N_A \cdot k_{vol} \cdot k_{cyt} \cdot V_{L1210}$$

$$= 0.12 \text{ µM} \cdot 6.022 \cdot 10^{23} \frac{1}{mol} \cdot 10^{-6} \frac{M}{\text{µ}M} \cdot 0.75 \cdot 0.63 \cdot 10^{-12}\, L = 34145,$$

where $N_A = 6.022 \cdot 10^{23} \frac{1}{mol}$ is the Avogadro constant, $V_{L1210} = 0.63 \cdot 10^{-12}\, L$ is the average cell volume of the L1210 cell line [19] and $k_{vol} = 10^{-6} \frac{M}{\text{µ}M}$, $k_{cyt} = 0.75$ are two scaling factors. The first is used to transform the concentration from µM to M; the second is introduced because only the reactions occurring in cytoplasm have been considered, that is, in the 75% of the total cell volume[20].

After the translation of concentrations and Michaelis-Menten constants to number of molecules, the propensities $a_j(x)$ for all reactions R_j were calculated. For the reactions formulated in terms of Michaelis-Menten or Hill kinetics, the propensities are computed by the same functions (see *Section Equations*) where parameters are expressed in terms of number of molecules, e.g. the propensity for the reaction catalyzed by DHFR is

$$a_{DHFR}(\#DHF, \#NADPH) = \frac{V_{max}^{\#} \;\; \#DHF \;\; \#NADPH}{\left(K_{DHF}^{\#} + \#DHF\right)\left(K_{NADPH}^{\#} + \#NADPH\right)},$$

where $K_{DHF}^{\#}$, $K_{DHF}^{\#}$ and $V_{max}^{\#}$ indicate the transformed Michaelis-Menten kinetic parameters.

In the case of the two mass-action reactions modeling the binding/unbinding of 5mTHF and SHMT the propensities are:

$$a_{binding}(\#5mTHF, \#SHMT) = k_{binding}/V_{L1210}\; \#5mTHF\; \#SHMT\,,$$

$$a_{unbinding}(\#5mTHF{:}SHMT) = k_{unbinding}\; \#5mTHF{:}SHMT\,.$$

Finally,

$$a_0(x) = \sum_{R_j} a_j(x)$$

gives the sum of all propensities in the current state x.

1.3 Calculations of dTMP synthesis capacity in mammals and yeast

The capacity of mammalian cells and yeast cells to synthesize sufficient levels of dTMP for DNA replication during S-phase was calculated using results from the model and other values from the literature, as discussed in the manuscript (see also Table 12). Detailed calculations are listed below.

Rate of dTMP synthesis in MTHFR CC case based on the computational model:

$$263.4\ \frac{\mu M}{h}\cdot\frac{1}{3600s}=0.073\ \frac{\mu M}{s}=4.38\ \frac{\mu M}{min}=3.51\ \cdot 10^{-6}\frac{pmol}{min}$$

by assuming a cell volume of $800\ \mu m^3\ =8\cdot 10^{-13}\ L$

dTMP synthesis needs required for replication:

Based on 59% of bp in human genome being AT [21]

$$3\cdot 10^9\ bp\ \cdot 0.59=1.77\ \cdot 10^9\ T\ molecules=2.94\ \cdot 10^{-15}\ mol\ T$$

are required.

Assuming furthermore 8 hour replication time in human ES cells or L1210 cells,

$$3.67\ \cdot 10^{-16}\ \frac{mol\ T}{h}\ =1.02\ \cdot 10^{-19}\ \frac{mol\ T}{s}$$

synthesis is required.

Assuming ES cell volume of $800\ \mu m^3\ =0.8\ pL=8\cdot 10^{-13}L$ the rate of dTMP synthesis required for faithful cell replication is

$$\frac{1.02\ \cdot 10^{-19}\frac{mol\ T}{s}}{8\ \cdot 10^{-13}L}=1.28\ \cdot 10^{-7}\ M\ \frac{T}{s}=0.13\ \mu M\frac{T}{s}=7.8\ \mu M\frac{T}{min}$$

($0.043\ \mu M\frac{T}{s}$ considering 24 hour cell doubling).

Ratio of T produced / T needed: 0.56

Rate of dTMP synthesis in *S. cerevisiae*:

44.8 µU TS activity/10^8 haploid cells; 1 µU = 1 pmol T/min; generation time = 150 minutes [22]

dTMP synthesis required for replication:

By considering the diploid genome with 61.5 % AT base pairs

$$12{,}156{,}677\ bp\ \cdot 0.615=7.5\ \cdot 10^6\ T\ molecules$$

are required.

We assume 150 minute generation time:

$$\frac{7.5\cdot 10^6}{150}\cdot\frac{\frac{T}{min}}{diploid\ cell}\cdot\frac{1}{6.02\cdot 10^{23}}\frac{mol}{molecules}=8.3\ \cdot 10^{-20}\frac{\frac{mol}{min}}{diploid\ cell}=8.3\ \cdot 10^{-8}\frac{\frac{pmol}{min}}{diploid\ cell}$$

If we assume that S-phase is 1/3 of cell cycle (50 min), then

$$8.3 \cdot 10^{-8} \frac{\frac{pmol}{min}}{diploid\ cell} \cdot 3 = 2.5 \cdot 10^{-7} \frac{\frac{pmol}{min}}{diploid\ cell} = 0.5 \frac{\frac{\mu mol}{min}}{haploid\ cell}$$

is needed to replicate the genome.

dTMP production as measured by ^{3}H-thymidine incorporation:

$$\frac{44.8\ \ \mu U\ TS\ activity}{10^8\ \ haploid\ cell} = \frac{44.8 \frac{pmol}{min}}{5 \cdot 10^7\ diploid\ cell} = 8.96 \cdot 10^{-7} \frac{\frac{pmol}{min}}{diploid\ cell} = 1.8 \frac{\frac{\mu mol}{min}}{haploid\ cell}\ .$$

References

1. Gostner R, Baldacci B, Morine MJ, Priami C. Graphical Modeling Tools for Systems Biology. *ACM Comput Surv*. 2015;47(2):1-21. doi:10.1145/2633461.

2. Scotti M, Stella L, Shearer EJ, Stover PJ. Modeling cellular compartmentation in one-carbon metabolism. *Wiley Interdiscip Rev Syst Biol Med*. 2013;5(3):343-365. doi:10.1002/wsbm.1209.

3. Nijhout HF, Reed MC, Budu P, Ulrich CM. A mathematical model of the folate cycle. *J Biol Chem*. 2004;279(53):55008-55016. doi:10.1074/jbc.M410818200.

4. Reed MC, Nijhout HF, Sparks R, Ulrich CM. A mathematical model of the methionine cycle. *J Theor Biol*. 2004;226(1):33-43. doi:10.1016/j.jtbi.2003.08.001.

5. Reed MC, Nijhout HF, Neuhouser ML, et al. A Mathematical Model Gives Insights into Nutritional and Genetic Aspects of Folate-Mediated One-Carbon Metabolism. *J Nutr*. 2006;136(10):2653-2661.

6. Reed MC, Thomas RL, Pavisic J, James SJ, Ulrich CM, Nijhout HF. A mathematical model of glutathione metabolism. *Theor Biol Med Model*. 2008;5(8). doi:10.1186/1742-4682-5-8.

7. Ulrich CM, Neuhouser M, Liu AY, et al. Mathematical modeling of folate metabolism: Predicted effects of genetic polymorphisms on mechanisms and biomarkers relevant to carcinogenesis. *Cancer Epidemol Biomarkers Prev*. 2008;17(7):1822-1831. doi:10.1161/CIRCULATIONAHA.111.087940.The.

8. Nijhout HF, Gregory JF, Fitzpatrick C, et al. A Mathematical Model Gives Insight into the Effects of Vitamin B-6 Deficiency on 1-Carbon and Glutathione Metabolism. *J Nutr*. 2009;139(4):784-791.

9. Reed MC, Gamble M V., Hall MN, Nijhout HF. Mathematical analysis of the regulation of competing methyltransferases. *BMC Syst Biol*. 2015;9(69). doi:10.1186/s12918-015-0215-6.

10. Matthews RG, Ghose C, Green JM, Matthews KD, Bruce Dunlap R. Folylpolyglutamates as substrates and inhibitors of folate-dependent enzymes. *Adv Enzyme Regul*. 1987;26:157-171. doi:10.1016/0065-2571(87)90012-4.

11. Stover P, Schirch V. 5-Formyltetrahydrofolate Polyglutamates Are Slow Tight Binding Inhibitors of Serine Hydroxymethyltransferase. *J Biol Chem*. 1991;266(3):1543-1550.

12. Jackson RC, Harrap KR. Studies with a Mathematical of Folate Metabolism. *Arch Biochem*. 1973;(16):827-841.

13. Morrison PF, Allegra CJ. Folate cycle kinetics in human breast cancer cells. *J Biol Chem*. 1989;264(18):10552-10566. http://www.ncbi.nlm.nih.gov/pubmed/2732237.

14. Seither RL, Trent DF, Mikulecky DC, Rape TJ, Goldman ID. Folate-pool interconversions and inhibition of biosynthetic processes after exposure of L1210 leukemia cells to antifolates. *J Biol Chem*. 1989;264(29):17016-17023.

15. Nijhout HF, Reed MC, Ulrich CM. Chapter 2 Mathematical Models of Folate-Mediated One-Carbon Metabolism. *Vitam Horm*. 2008;79:45-82. doi:10.1016/S0083-6729(08)00402-0.

16. Stover PJ, Field MS. Trafficking of Intracellular Folates. *Adv Nutr*. 2011;2:325-331. doi:10.3945/an.111.000596.325.

17. Pawelek PD, Allaire M, Cygler M, MacKenzie RE. Channeling efficiency in the bifunctional methylenetetrahydrofolate dehydrogenase/cyclohydrolase domain: The effects of site-directed mutagenesis of NADP binding residues. *Biochim Biophys Acta - Protein Struct Mol Enzymol*. 2000;1479:59-68. doi:10.1016/S0167-4838(00)00058-3.

18. Kim DW, Huang T, Schirch D, Schirch V. Properties of Tetrahydropteroylpentaglutamate Bound to 10-Formyltetrahydrofolate Dehydrogenase. *Biochemistry*. 1996;35:15772-15783.

19. Aherne GW, Ward E, Lawrence N, et al. Comparison of plasma and tissue levels of ZD1694 (Tomudex), a highly polyglutamatable quinazoline thymidylate synthase inhibitor, in preclinical models. *Br J Cancer*. 1998;77(2):221-226. <Go to ISI>://000071496600007.

20. Luby-Phelps K. Cytoarchitecture and Physical properties of cytoplasm: Volume, Viscosity, Diffusion, Intracellular Surface Area. *Int Rev Cytol*. 2000;192:189-221.

21. Antonarakis ES. Human Genome Sequence and Variation. In: Speicher MR, ed. *Vogel and Motulsky's Human Genetics: Problems and Approaches*. Springer-Verlag berlin, Heidelberg; 2010:31-53.

22. Greenwood MT, Calmels EM, Storms RK. Growth-rate-dependent regulation of the expression and inactivation of thymidylate synthase in Saccharomyces cerevisiae. *J Bacteriol*. 1986;168(3):1336-1342.

23. Strong B, Tendlers SJ, Seither L, David I. Purification and Properties of Serine Hydroxymethyltransferase Synthase from L1210 Cells. *J Biol Chem*. 1990;265(21):12149-12155.

24. Thorndike J, Gaumont Y, Kisliuk RL, et al. Inhibition of glycinamide ribonucleotide formyltransferase and other folate enzymes by homofolate polyglutamates in human lymphoma and murine leukemia cell extracts. *Cancer Res*. 1989;49:158-163.

25. Rayl E a, Moroson B a, Beardsley GP. The Human purH Gene Product , 5-Aminoimidazole-4-carboxamide Ribonucleotide Formyltransferase / IMP Cyclohydrolase. *J Biol Chem*. 1996;271(4):2225-2233. doi:10.1074/jbc.271.4.2225.

26. Matthews RG, Ross J, Baugh CM, Cook JD, Davis L. Interactions of pig liver serine hydroxymethyltransferase with methyltetrahydropteroylpolyglutamate inhibitors and with tetrahydropteroylpolyglutamate substrates. *Biochemistry*. 1982;21:1230-1238.

27. Manieri W, Moore ME, Soeller MB, Tsang P, Caperelli CA. Human Glycinamide Ribonucleotide Transformylase: Active Site Mutants as Mechanistic Probes. *Biochemistry*. 2007;46(1):156-163. doi:10.1038/jid.2014.371.

28. Strong W, Schirch V. In vitro conversion of formate to serine: effect of tetrahydropteroylpolyglutamates and serine hydroxymethyltransferase on the rate of 10-formyltetrahydrofolate synthetase. *Biochemistry*. 1989;28(24):9430-9439.

29. Radparvar S, Houghton PJ, Houghton J a. Characteristics of thymidylate synthase purified from a human colon adenocarcinoma. *Arch Biochem Biophys*. 1988;260(1):342-350. http://www.ncbi.nlm.nih.gov/pubmed/3341747.

Supplementary Figures

Figure S1. The reaction-based specification of the model according to the notation introduced in [1] (the same Figure is also provided in the main text). Rectangles identify model variables, non-boxed substrates are model constants, green circles identify enzymes, dark blue arcs identify matter transformation, and light blue arcs identify regulations (dotted lines indicate promotions and solid lines indicate inhibitions). The two boxes indicate the affiliation of the reactions and variables to the folate cycle and the homocysteine remethylation cycle, respectively.

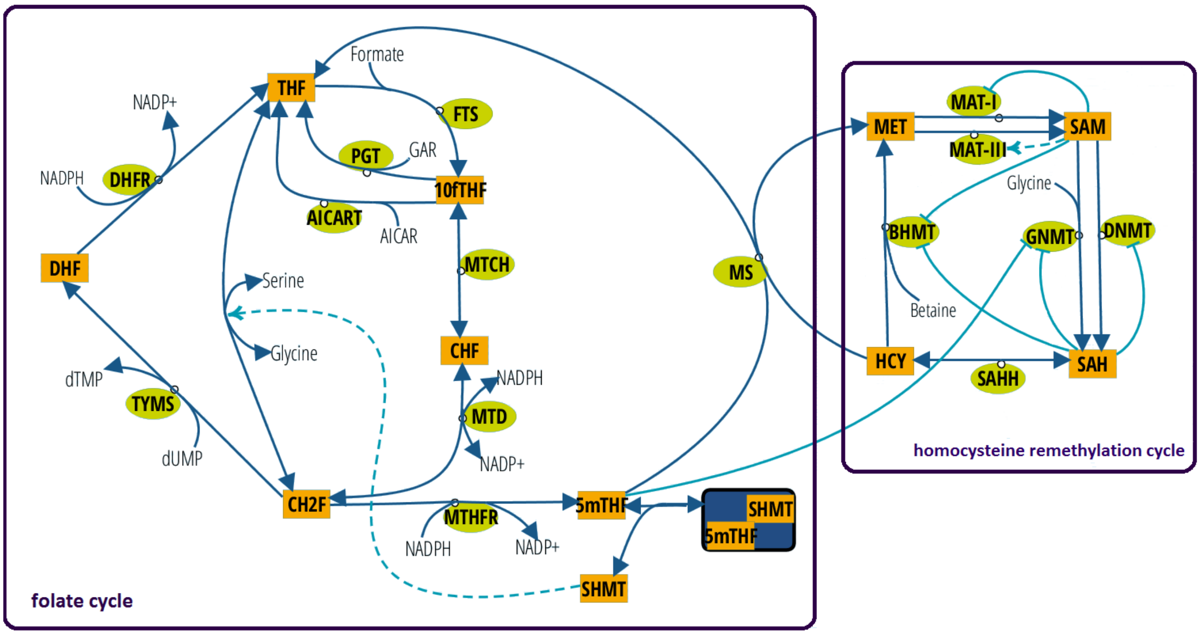

Figure S2. A point-to-point comparison between the herein proposed model and the one in [5]. Rectangles indicate variables of the model; enzymes are indicated in oval frames attached to the corresponding reaction represented by arcs; non-boxed substrates/products are model constants. Based on the model structure used in [5] the figure displays the applied modifications using different colors. Green colored reactions as well as green framed variables and enzymes indicate the adopted parts of the model. Orange colored reactions as well as orange framed variables and enzymes indicate parts of the network which have been modified or added. Black arcs and black framed variables and enzymes indicate parts of the network we did not consider. The comparison considers the structure of model equations, while parameter estimates have been updated with respect to [5] according to Table S4. The figure has been adapted from [5].

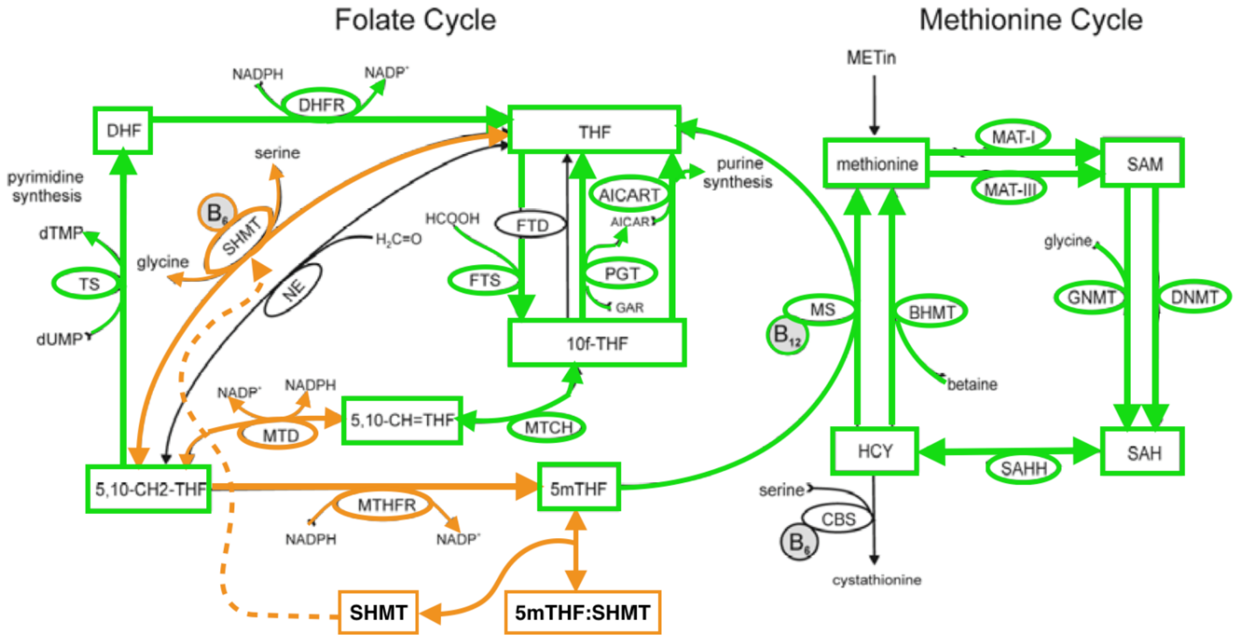

Supplementary Tables

Table S1. List of Abbreviations and acronyms.

5mTHF	5-methyl tetrahydrofolate
10fTHF	10-formyltetrahydrofolate
AICAR	5-Aminoimidazole-4-carboxamide ribonucleotide
AICARFT	Phosphoribosylaminoimidazolecarboxamide formyltransferase
BHMT	Betaine-homocysteine methyltransferase
CHF	5,10-methenyltetrahydrofolate
CH2F	5,10-methylenetetrahydrofolate
DHF	Dihydrofolate
DHFR	Dihydrofolate reductase
DNMT	DNA methyltransferase
dUMP	Deoxyuridine monophosphate
dTMP	Deoxythymidine monophosphate
FOCM	Folate-mediated one-carbon metabolism
FTD	10-formyltetrahydrofolate dehydrogenase
FTS	Formate-tetrahydrofolate ligase
GAR	Glycinamide ribonucleotide
GNMT	Glycine N-methyltransferase
HCY	Homocysteine
MAT-I	Methionine adenosyltransferase 1
MAT-III	Methionine adenosyltransferase 3
MET	Methionine
MTCH	Methenyltetrahydrofolate cyclohydrolase
MTD	Methylenetetrahydrofolate dehydrogenase

MTHFD	Methylenetetrahydrofolate dehydrogenase
MTHFR	Methylenetetrahydrofolate reductase
MTR	Methionine synthase
NADP+	Nicotinamide adenine dinucleotide phosphate
NADPH	Reduced form of Nicotinamide adenine dinucleotide phosphate
PGT	Phosphoribosylglycinamide formyltransferase
THF	Tetrahydrofolate
TYMS	Thymidylate synthase
SAH	S-adenosyl-homocysteine
SAHH	S-adenosylhomocysteine hydrolase
SAM	S-adenosyl-methionine
SHMT	Serine Hydroxymethyltransferase
SUMO	Small ubiquitin-like modifier

Table S2. Initial concentrations of the twelve model variables.

Substrate/Enzyme	μM	Reference
THF	4.61	[6]
10fTHF	3.41	[6]
CHF	0.28	[6]
CH2H	0.51	[6]
DHF	0.039	[6]
5mTHF free	4.5	[8]
5mTHF:SHMT	4.5	[8]
SHMT free	4.5	[8]
HCY	1.12	[6]
MET	49.2	[6]
SAM	81.1	[6]
SAH	19.1	[6]

Table S3. Concentrations of the constant substrates included in the model.

Constant Substrate	μM	Cell line	Reference
NADPH	58	L1210	[14]
NADP+	18	L1210	[14]
dUMP	20	L1210	[14]
Serine	468	L1210	[14]
Glycine	1850	L1210	[14]
GAR	10	L1210	[14]
AICAR	2.1	L1210	[14]
Formate	200	L1210	[23]
Betaine	50	--	[6]

Table S4. Model parameter estimates for the folate cycle grouped by reactions. All concentrations are expressed in μM, while time is expressed in hours. For each value the reference cell line and the length of the glutamate chain of the associated folate are also indicated when available in literature.

Parameter	Metabolite	Value	Length of glutamate chain	Cell line	Reference
$R_{AICARFT}: 10fTHF \rightarrow THF$					
V_{max}		63350		MCF-7	[13]
K_m	10fTHF	0.3	4-6	Human leukemia	[24]
K_m	AICAR	16.8		Human purH	[25]
$R_{DHFR}: DHF \rightarrow THF$					
V_{max}		22200		L1210	[14]
K_m	DHF	0.5		L1210	[14]
K_m	NADPH	4.3		L1210	[14]
$R_{FTS}: THF \rightarrow 10fTHF$					
V_{max}		45900	6	L1210	[23]
K_m	THF	0.1	5	L1210	[23]
K_m	formate	16	5	L1210	[23]
$R_{MTCH}: CHF \rightarrow 10fTHF$					
V_{max}		2916000	5	L1210	[23]
K_m	CHF	4	5	L1210	[23]
$R_{MTCH}: 10fTHF \rightarrow CHF$					
V_{max}		2916000	5	L1201	[23]
K_m	10fTHF	20		L1210	[23]
$R_{MTD}: CHF \rightarrow CH2F$					
V_{max}		594000	5/6	L1210	[23]
K_m	CHF	6.3		Human DC301	[17]

K_m	NADPH	10.5		Human DC301	[17]
R_{MTD} : $CH2F \rightarrow CHF$					
V_{max}		594000	5/6	L1210	[23]
K_m	CH2F	2	5	L1210	[23]
K_m	NADP+	2	5	L1201	[23]
R_{MTHFR} : $CH2F \rightarrow 5mTHF$					
V_{max}		120	5	Pig liver	[26]
K_m	CH2F	0.26	5	Pig liver	[26]
K_m	NADPH	125	5	Pig liver	[26]
R_{MTR} : $5mTHF + HCY \rightarrow THF + MET$					
V_{max}		30			This manuscript, estimated in the range 0.024[10] - 500[6] μM/h
K_m	5mTHF	0.5	6	Pig liver	[10]
K_m	HCY	0.1			[5]
R_{PGT} : $10fTHF \rightarrow THF$					
V_{max}		6600			[5]
K_m	10fTHF	0.9		human	[27]
K_m	GAR	1.1		human	[27]
R_{SHMT} : $THF \rightarrow CH2F$					
K_m	Serine	600	5	L1210	[23]
K_m	THF	0.2	5/6	L1210	[23]
k_{cat}		18000	5/6	L1210	[23]
R_{SHMT} : $CH2F \rightarrow THF$					
K_m	Glycine	3000		L1210	[23]
K_m	CH2F	0.2		L1210	[23]
k_{cat}		45000	4	Rabbit liver	[28]

R_{TYMS} : $CH2F \rightarrow DHF$					
V_{max}		4200		L1210	[14]
K_m	CH2F	4.3	1	Human colon	[29]
K_m	dUMP	3.6	1	Human colon	[29]
(un-)binding of 5mTHF and SHMT					
$k_{unbinding}$		1980	3	Rabbit liver	[11]
$k_{binding}$		7200	3	Rabbit liver	[11]

Table S5. Model parameter estimates for the homocysteine remethylation cycle grouped by reactions. All concentrations are expressed in µM, while time is expressed in hours.

Parameter	Metabolite	Value	Reference
R_{BHMT} : $HCY \rightarrow MET$			
V_{max}		2160	[6]
K_m	HCY	12	[6]
K_m	Betaine	100	[6]
R_{DNMT} : $SAM \rightarrow SAH$			
V_{max}		180	[6]
K_m	SAM	1.4	[6]
K_i	Inhibition by SAH	1.4	[6]
R_{GNMT} : $SAM \rightarrow SAH$			
V_{max}		245	[6]
K_m	SAM	32	[6]
K_m	Glycine	130	[6]
K_i	Inhibition by SAH	18	[6]

R_{MAT-I} : $MAT \rightarrow SAM$			
V_{max}		260	[6]
K_m	MET	41	[6]
$R_{MAT-III}$: $MAT \rightarrow SAM$			
V_{max}		220	[6]
K_m	MET	300	[6]
K_a	Activation by SAM	360	[6]
R_{SAHH} : $SAH \rightarrow HCY$			
V_{max}		320	[6]
K_m	SAH	6.5	[6]
R_{SAHH} : $HCY \rightarrow SAH$			
V_{max}	HCY -> SAH	4530	[6]
K_m	HCY	150	[6]

Table S6. Stochastic propensities for all model reactions. Reactions are indicated by the enzyme catalyzing them. The propensities are calculated in the model steady state by considering the CC and the TT case of the MTHFR polymorphism. Comparison between the two scenarios is provided by their difference expressed in % of CC.

	FTS	MTCH		MTD		AICARFT	PGT
		10fTHF → CHF	CHF → 10fTHF	CHF → CH2F	CH2F → CHF		
CC	6,596,297,718	215,116,909,117	211,683,279,282	25,562,411,625	22,102,671,431	1,920,578,414	1,499,148,516
TT	11,313,005,226	285,290,029,076	277,499,278,768	34,615,583,310	26,918,068,257	1,947,149,894	1,558,090,684
Difference (% of CC)	71.5	32.6	31.1	35.4	21.8	1.4	3.9
	MTHFR	DHFR	TYMS	SHMT		Binding of 5mTHF & SHMT	Unbinding of 5mTHF & SHMT
				CH2F → THF	THF → CH2F		
CC	6,132,514	115,312,264	74,213,168	1,260,072,867,375,039	344,862,817,955,790	4,248,251,645	4,259,314,801
TT	2,023,259	115,312,264	92,071,952	5,397,444,475,851,732	3,193,367,766,042,925	1,865,166,039	1,864,858,7298
Difference (% of CC)	-67.0	0.0	24.1	328.3	826.0	-56.1	-56.2

	BHMT	MAT-I	MAT-III	GNMT	DNMT	SAHH		MTR
						SAH → HCY	HCY → SAH	
CC	4,5761,513	34,996,581	58,188,162	107,600,905	24,807,058	8,1150,893	29,144,522	6,158,799
TT	49,927,635	36,950,816	51,905,890	239,168,069	9,876,025	84,535,820	32,582,932	2,017,561
Difference (% of CC)	9.1	5.7	-10.8	122.3	-60.2	4.2	11.8	-67.2
